# Hatching Asynchrony in Oviraptorid Dinosaurs Sheds Light on Their Unique Nesting Biology

**DOI:** 10.1093/iob/obz030

**Published:** 2019-11-22

**Authors:** T -R Yang, T Engler, J N Lallensack, A Samathi, M Makowska, B Schillinger

**Affiliations:** 1 Section of Paleontology, Institute of Geosciences, Rheinische Friedrich-Wilhelms-Universität Bonn, Nussallee 8, 53115 Bonn, Germany; 2 Division of Geology, National Museum of Natural Science, Guancian Road 1, 40453 Taichung, Taiwan; 3 Biodiversity and Conservation Research Unit. Walai Rukhavej Botanical Research Institute, Mahasarakham University, 44150 Maha Sarakham, Thailand; 4 Photons for Engineering and Manufacturing Group, Paul Scherrer Institute, Forschungsstrasse 111, 5232 Villigen PSI, Switzerland; 5 Heinz Maier-Leibnitz Zentrum (MLZ), Lichtenbergstraße 1, 85748 Garching, Germany

## Abstract

Dinosaur nesting biology has been an intriguing research topic, though dinosaur behaviors were relatively less illuminated because of the constraints of the fossil record. For instance, hatching asynchrony, where eggs in a single clutch hatch at different times, is unique to modern neoavian birds but was also suggested to be present in oviraptorid dinosaurs based on a possible partial clutch of four embryo-containing eggs from Mongolia. Unfortunately, unequivocal evidence for the origination of these eggs from a single clutch is lacking. Here we report a new, better preserved partial oviraptorid clutch with three embryo-containing eggs—a single egg (Egg I) and a pair (Egg II/III)—from the Late Cretaceous Nanxiong Group of Jiangxi Province, China. Geopetal features indicate that the pair of eggs was laid prior to the single egg. Neutron tomographic images in combination with osteological features indicate that the embryo of the single egg is less developed than those of the paired eggs. Eggshell histology suggests that the embryo-induced erosion in the paired eggs is markedly more pronounced than in the single egg, providing a new line of evidence for hatching asynchrony. The inferred hatching asynchrony in combination with previously surmised thermoregulatory incubation and communal nesting behaviors very likely suggests that oviraptorid dinosaurs presented a unique reproductive biology lacking modern analogs, which is contrary to the predominant view that their reproductive biology was intermediate between that of modern crocodiles and birds.

## Synopsis

A biologia de nidificação de dinossauros tem sido um tópico de pesquisa intrigante, embora o comportamentos do dinossauros tenham sido relativamente menos compreendidos devido às restrições do registro fóssil. Por exemplo, eclosão assincrônica, onde os ovos em uma única ninhada eclodem em momentos diferentes, é exclusiva em aves neoavianas modernas, tem-se sugerido estar presente em dinossauros oviraptorídeos com base em uma possível ninhada parcial de quatro ovos da Mongólia contendo embriões. Infelizmente, faltam evidências inequívocas para a origem desses ovos a partir de uma única embreagem. Relatamos aqui uma nova parcial ninhada oviraptoroide melhor preservada, com três ovos contendo embriões—um único ovo (ovo I) e um par (ovo II/III)—do Grupo de Nanxiong Cretáceo Final da Província de Jiangxi, China. A erosão induzida pelo embrião nos ovos emparelhados é significativamente maior que a do ovo único, fornecendo uma evidência inequívoca de eclosão assincrônica. A inferida de eclosão assincrônica, em combinação com as linhas de evidência anteriores, sugere que os dinossauros oviraptorídeos apresentavam uma biologia reprodutiva muito singular, sem análogos modernos, contrariando a visão predominante de que sua biologia reprodutiva era intermediária entre a dos crocodilos e pássaros modernos. Translated to Portuguese by Diego Vaz (dbistonvaz@vims.edu)

恐龍的築巢生物學因為化石紀錄的限制, 很少有相關的進展。但恐龍的生殖生 物學一直是一個迷人的研究議題。舉例來說, 不同步孵化（同一窩蛋在不同時 間點孵化）是現生鳥類獨有的特徵, 然而曾有研究報導了發現自蒙古的四顆含 竊蛋龍胚胎的恐龍蛋, 認為它們可能來自同一窩蛋, 且這四個胚胎的不同發育 程度指示了不同步孵化。然而, 這四顆蛋是否來自同一窩蛋卻仍然缺乏確切的 證據。本研究報導一件產自中國江西晚白堊世地層的三顆竊蛋龍胚胎蛋（包含 同一次產卵所下的一對蛋與另外一顆蛋）標本。一些指示地層上下的地質特徵 顯示, 對蛋的產下時間是早於單顆蛋的。X光斷層掃描分析與骨骼學的特徵共 同指示出, 單顆蛋內的胚胎的發育程度少於對蛋內的胚胎。蛋殼微組織學上, 可觀察到對蛋的蛋殼有較單顆蛋嚴重的胚胎侵蝕痕跡。這些證據都支持了竊蛋 龍的不同步孵化。此外, 結合前人研究所提出的竊蛋龍孵蛋與共巢行為假說, 我們認為, 竊蛋龍的生殖生物學, 並非單純只是介於恐龍與鳥之間的過渡型與 中間態, 而是完全異於現生生物、無可比擬且非常獨特的。 Translated to Chinese by T.-R. Yang (lereage@gmail.com)

Die Nistbiologie von Dinosauriern ist ein faszinierendes Forschungsfeld, obwohl ihr Nistverhalten wegen des lückenhaften Fossilberichts noch weitgehend im Dunkeln liegt. Beispielsweise ist asynchrones Schlüpfen, wo Eier eines Geleges zeitlich versetzt schlüpfen, einzigartig für moderne Vögel (Neoaves), wurde aber auch für oviraptoride Dinosaurier anhand eines möglichen Teilgeleges aus vier Eiern mit Embryonen aus der Mongolei vorgeschlagen. Eindeutige Belege, dass diese Eier tatsächlich zu einem einzigen Gelege gehören, fehlen jedoch. In der vorliegenden Arbeit berichten wir über ein neu entdecktes, besser erhaltenes Teilgelege aus drei Eiern mit Embryonen—ein einzelnes Ei (Ei I) sowie ein Paar (Ei II/III)—aus der oberkretazischen Nanxiong-Gruppe der chinesischen Provinz Jiangxi. Geopetalgefüge zeigen, dass das Ei-Paar vor dem einzelnen Ei gelegt wurde. Neutronen Computertomographische Bilder in Kombination mit osteologischen Merkmalen lassen vermuten, dass der Embryo des einzelnen Eis weniger entwickelt war als jene des Ei-Paares. Histologische Untersuchungen der Eischalen zeigen, dass die vom Embryo ausgehende Erosion in den gepaarten Eiern deutlich ausgeprägter war als in dem einzelnen Ei, was eine neue Evidenzlinie für asynchrones Schlüpfen darstellt. Das asynchrone Schlüpfen, in Kombination mit der zuvor vorgeschlagenen thermoregulatorischen Bebrütung und kommunales Nisten, deutet auf eine einzigartige Reproduktionsbiologie oviraptorider Dinosaurier, die ohne modern Analogien ist. Dies widerspricht der vorherrschenden Sichtweise, die Reproduktionsbiologie von Oviraptoriden sei intermediär zwischen der moderner Krokodile und jener von Vögeln gewesen. German translated by J. N. Lallensack (jens.lallensack@gmail.com)

## Background

### Hatching asynchrony in reptiles, birds, and dinosaurs

**Fig. 1:**
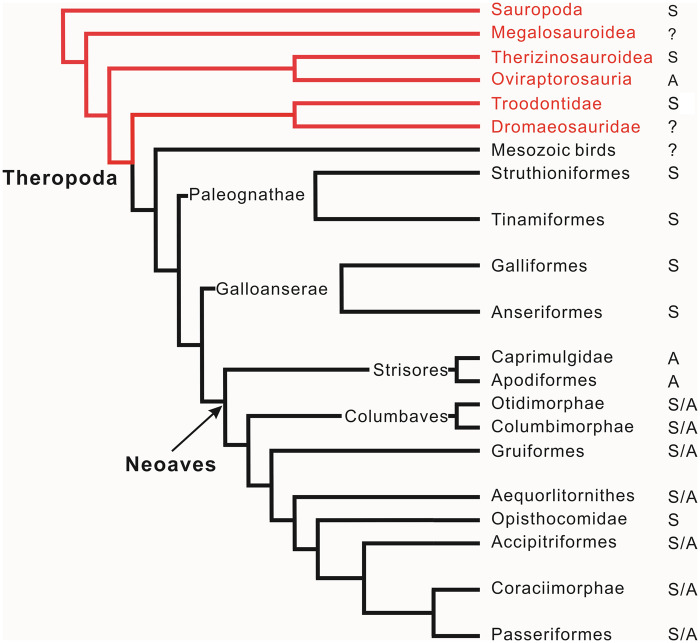
Distribution of hatching modes (S, synchronous; A, asynchronous) among saurischian dinosaurs based on the fossil record for non-avian dinosaurs and on ornithological observations for birds (Stoleson and Beissinger 2010). The phylogeny was compiled by integrating the avian phylogeny (black) based on Prum et al. (2015) and a general dinosaur phylogeny (red) based on Varricchio and Jackson (2016).

Hatching asynchrony, which is only found in extant birds (Stoleson and Beissinger 2010; Prum et al. 2015), occurs when incubation of the eggs commences before the clutch is finished by one or several females (Lee and Lima 2017; Fig. 1). In complete synchrony, incubation begins after the clutch is finished and all eggs hatch at the same time. However, in complete asynchrony, incubation begins when the first egg is laid, and all eggs hatch gradually over a period of time. Some birds show partial synchrony (or partial asynchrony), in which incubation begins after several ovipositions. Non-avian reptile eggs hatch synchronously because they are laid *en masse* and develop simultaneously. A study discovered inter-embryo communication between siblings in the same turtle clutch, coordinating the pace of their development in order to hatch together (McGlashan et al. 2012). This mechanism was also observed in snakes (Aubret et al. 2016) and, albeit speculative, probably also existed in those dinosaurs that laid a whole clutch simultaneously and buried their eggs without incubation and parental care. This was likely the case in the basal sauropodomorph *Massospondylus* (Reisz et al. 2012), sauropods (Chiappe et al. 1998; Sander et al. 2008), lambeosaurine hadrosaurs (Horner 1999), and therizinosaurs (Kobayashi et al. 2013).

Among non-avian dinosaurs, titanosaurs, oviraptorosaurs, and troodontids yield the most abundant fossil record of eggs and embryos (Norell et al. 1994, 2001; Varricchio et al. 1997, 1999, 2002; Chiappe et al. 1998, 2001; Cheng et al. 2008; Weishampel et al. 2008; Shao et al. 2014). Previous research has suggested that sauropods buried their eggs in the substrate based on shell porosity analyses of sauropod eggs from France, Spain, and India (Seymour 1979; Sander et al. 1998, 2008; Deeming 2006). Chiappe et al. (2004) studied six egg clutches from Auca Mahuevo, Argentina, and interpreted them as open nests of a sauropod dinosaur based on lithologic relations (Chiappe et al. 2004). Furthermore, it was suggested that the Auca Mahuevo titanosaur eggs were incubated in high humidity conditions (Grellet-Tinner et al. 2004; Grellet-Tinner et al. 2006).

Jackson et al. (2008) and Sander et al. (2008) demonstrated that the Auca Mahuevo titanosaur eggs have low porosity, thus indicating that these dinosaurs did not bury their eggs (open nesting), again echoing Chiappe et al.’s (2004) interpretation. Hechenleitner et al. (2015) argued against the “open nesting hypothesis” by proposing that titanosaurs adopted mound nesting or burrow nesting. While no fossils similar to the gravid oviraptorosaur described by Sato et al. (2005) have been reported for sauropods, it is most likely that sauropods deposited clutches in one event and that all eggs hatched synchronously without incubation by adults (Fig. 1; Chiappe et al. 2004; Jackson et al. 2008; Sander et al. 2008; Ruxton et al. 2014). Such a hatching pattern shows great similarity to modern crocodiles and thus very likely represents the plesiomorphic condition in dinosaurs.

The reproductive biology of troodontid dinosaurs was illuminated by embryo-bearing eggs, an adult-associated clutch, and several egg clutches (Varricchio et al. 1997, 1999). Previous studies based on these fossils suggested brooding and polygamous behaviors in troodontid dinosaurs and that they represent an intermediate stage between oviraptorids and modern birds (Fig. 1; Varricchio and Jackson 2016).

Oviraptorids left behind the most abundant record of fossil dinosaur eggs and clutches. Important insights into dinosaur reproduction biology have been gained from two gravid females (Sato et al. 2005; He et al. 2012), numerous embryo-containing eggs (Chiappe et al. 1998, 2001; Norell et al. 2001; Cheng et al. 2008; Weishampel et al. 2008; Wang et al. 2016), and several clutch-adult associations (Norell et al. 1995, 2018; Dong and Currie 1996; Clark et al. 1999; Fanti et al. 2012). Most oviraptorid embryo-containing eggs were discovered without siblings from the same clutch. In 2008, four partial eggs with embryonic remains (two of which adhere to each other) from the Nemegt Formation at Bugin-Tsav, in the southwestern Gobi Desert of Mongolia, were reported, providing insights into hatching asynchrony in oviraptorid dinosaurs (Weishampel et al. 2008). However, poor preservation precluded attribution of the four embryo-containing eggs to a single clutch. Although Weishampel et al. (2008) claimed that the four eggs were found as an aggregate of fragments in a circular depression, the interpretation that they were from the same clutch remained controversial because of the absence of evidence for direct associations.

Although the study by Weishampel et al. (2008) improved our understanding of the reproductive biology of oviraptorid dinosaurs, the poor preservation of the material led to uncertainty about the assumed hatching asynchrony. This study furthermore aims to provide further insights into oviraptorosaur reproductive biology based on the new specimen.

### Embryology of oviraptorids

Individual embryo-containing eggs have contributed to our understanding of dinosaur embryology; however, paleontologists have been unable to elucidate the entire developmental sequence of dinosaur embryos in the absence of multiple monotaxic embryos. At the Ukhaa Tolgod site of Mongolia, Norell et al. (1994) discovered a theropod embryo that displays several apomorphies of the Oviraptoridae, and possibly belongs to *Oviraptor philoceratops*. Later, four oviraptorid embryos *in ovo* from Bugin-Tsav were reported, potentially indicating hatching asynchrony in oviraptorids (Weishampel et al. 2008). Cheng et al. (2008) described two oviraptorid embryos (NMNS-0015276-F02-embryo-01 and CM-61) from the Nanxiong Group of Ganzhou Basin, Jiangxi Province, China, and assigned them to *Heyuannia huangi* based on eggshell microstructure and geographical affinity to previously described *H. huangi*. Wang et al. (2016) reported three isolated oviraptorid embryo-containing eggs from Nankang District, Ganzhou County, Jiangxi Province, China (IVPP V20182, IVPP V20183, and IVPP V20184). Although the eggshell microstructure supports the assignment to *H.huangi* (Wang et al. 2016), they assigned the embryos to Oviraptoridae *incertae sedis* because they did not show autapomorphies at species level. The study also described 20 osteological features that change substantially during ontogeny in oviraptorids. For instance, the distal caudal vertebrae change from being unossified in the immature stage to ossified in the mature stage. Subsequently, *Beibeilong sinensis*, one of the best preserved dinosaur embryos, was described, providing the first known association between skeletal remains and eggs of caenagnathids (Pu et al. 2017). These discoveries demonstrated the abundance of oviraptorid, or oviraptorosaurian, embryos in China and Mongolia (Norell et al. 1994; Cheng et al. 2008; Weishampel et al. 2008; Shao et al. 2014; Wang et al. 2016; Pu et al. 2017). Despite many reports of oviraptorid embryonic remains *in ovo*, the position of the embryos *in ovo* is unclear and poorly discussed. In Norell et al.’s (1994) report, the skull of the undisturbed embryo *in ovo* (IGM 100/971) sits near the blunt end, which is indicated by the node ornamentation of the eggshell. The *Beibeilong* embryo was discovered with another five eggs and the skull of the *Beibeilong* embryo points to the center of the clutch as indicated by the long axes of the other five eggs (Pu et al. 2017). However, the pose of an oviraptorid embryo *in ovo* has never been discussed (Norell et al. 1994; Cheng et al. 2008; Weishampel et al. 2008; Shao et al. 2014; Wang et al. 2016; Pu et al. 2017).

### Nesting biology of oviraptorids

A complete oviraptorid clutch consists of more than 30 eggs arranged in pairs which in turn are arranged in three to four superimposed rings (Yang et al. 2019). Pairing implies monoautochronic ovulation in oviraptorid dinosaurs, which was also inferred based on an oviraptorosaurian pelvis with a pair of eggs inside (Sato et al. 2005). The latter fossil and the paired arrangement in oviraptorid clutches render post-laying parental manipulation unlikely and indicate a sequential laying from the inner to the outer ring. Within the clutch, the long axis of the eggs is inclined at about 60° from the vertical toward the clutch center, with the blunt end pointing toward the clutch center (see Yang et al. 2019). These features highlight the peculiar nesting mode of oviraptorid dinosaurs and offer geopetal criteria that are of central importance for this study.

In addition to their peculiar nesting mode, oviraptorid dinosaurs were hypothesized to exhibit a number of behavioral characters unique to living birds. Norell et al. (1995) proposed brooding behavior in oviraptorid dinosaurs based on a report of the egg clutch-adult association in the oviraptorid dinosaur *Citipati osmolskae* from Mongolia. In this report, they only proposed “sitting behavior atop a clutch” but not “contact incubation behavior.”

Later discoveries of oviraptorid adult-associated clutches (*O.philoceratops*, *Nemegtomaia barsboldi*, cf. *Machairasaurus*, and *C.osmolskae*) further suggested that the behavior of sitting atop a clutch is a shared character of Oviraptoridae (Dong and Currie 1996; Clark et al. 1999; Fanti et al. 2012; Norell et al. 2018). However, an increasing number of studies tended to impose the “incubation behavior hypothesis” on derived feathered dinosaurs in the absence of a detailed examination of all available evidence. Yang et al. (2019) reported five complete oviraptorid nests excavated from Jiangxi Province, China, with more than 30 eggs, and found sediment interbedded between the stacked rings of eggs. This indicates that heat was difficult to transfer to the lower part of the clutch, which suggests that the “brooding” specimens could represent guarding instead of incubation behavior as shown by some extant birds. Deeming (2002) also pointed out that the mere close physical association of the *Oviraptor* adult with the eggs is insufficient for inferring brooding behavior (here he conflated brooding and incubation). Therefore, there is no consensus on the presence and mode of brooding behavior in non-avian dinosaurs.

Furthermore, Varricchio et al. (2008) proposed polygamous behavior in oviraptorid dinosaurs based on their ratio of clutch volume to adult mass that is closest to that seen in modern polygamous birds. Polygamous behavior is often associated with paternal care and communal nesting behaviors. Whereas the hypothesis of paternal care in oviraptorids is still in debate, the communal nesting behavior in oviraptorids was later supported by elemental analyses of eggshells from the same clutch that show inter-pair differences of phosphorus content disparity between the mammillary and continuous zones, which is an indicator of female age since younger females laid eggs with higher disparity of phosphorus between mammillary and continuous zones (Cusack et al. 2003; Yang et al. 2016). In this study, we will only discuss the possibility of communal nesting behavior since it is pertinent to our hypothesis. We attempt to test the above two hypotheses—brooding and communal nesting behaviors—and provide a comprehensive nesting biology model for oviraptorid dinosaurs.

## Material and methods

**Fig. 2:**
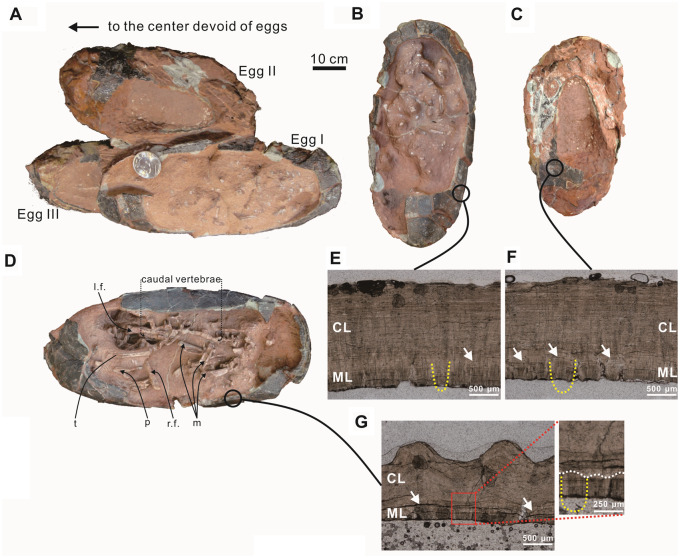
Hatching asynchrony inferred from a partial clutch containing three oviraptorid embryos *in ovo*. (**A**) Oviraptorid eggs (SMNH-20140105) containing embryological material before preparation and separation into the individual eggs. This specimen is shown in a field-top view. The arrow points to the center of the original clutch, which can be identified based on the blunt ends of the eggs. (**B**–**D**) Oviraptorid egg containing scattered embryonic remains after preparation (B: Egg I, SMNH-20140105-1; C: Egg II, SMNH-20140105-2; D: Egg III, SMNH-20140105-3) in a field-top view. (**E**–**G**) Photomicrograph of sectioned eggshells from (E) Egg I, (F) Egg II, (G) Egg III. The undulating boundary between the CL and ML, as marked by white arrows, is a distinct feature of *Macroolithus* sp. (eggs of oviraptorids). The mammillae were a cone-like structure as shown by yellow dash lines. The significant erosion of the mammillary layer shown by the absence of mammillary tips indicates calcium absorption of the developing embryo. i, ilium; l.f., left femur; mt, metatarsal; p: pubis; r.f., right femur; t: tibia; v, vertebrae.

A block of a partial clutch (SMNH-20140105; Fig. 2A) contains an individual egg (SMNH-20140105-1; Fig. 2B) and a pair of eggs (SMNH-20140105-2 and SMNH-20140105-3; Figs. 2C and D), both of which contain embryonic remains. Hereafter, the eggs will be termed Egg I (SMNH-20140105-1), Egg II (SMNH-20140105-2), and Egg III (SMNH-20140105-3) for ease of discussion.

**Fig. 3:**
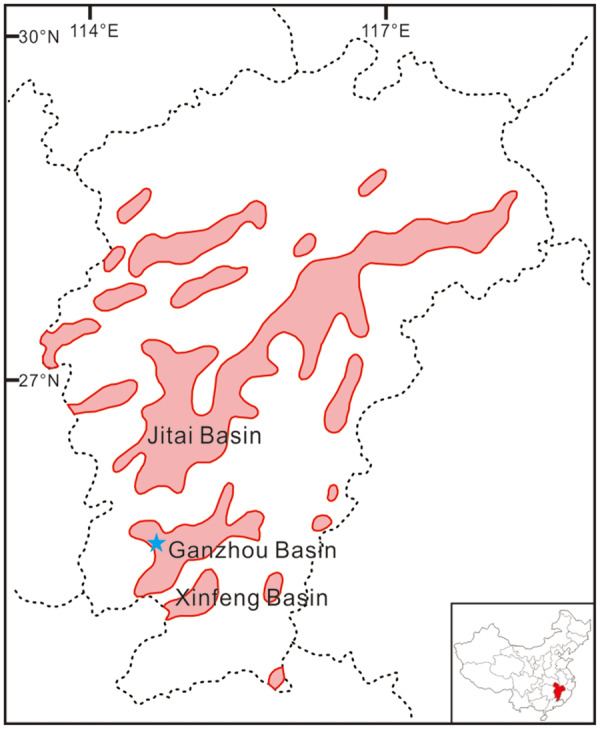
Map of Jiangxi Province, China, showing the distribution of Cretaceous red bed basins (red areas). The blue star indicates the locality where the studied specimen SMNH-20140105 was discovered.

The partial clutch was discovered as a single block in the Late Cretaceous Nanxiong Group, Ganzhou Basin, southern Jiangxi Province, China. Geographically, the site of discovery is located in the technological development zone near Dayu County of Ganzhou City. Geologically, the Ganzhou Basin is composed of two NE-SW striking sub-basins, including the northeastern part and the southwestern part (Fig. 3). Like most other red bed basins in Jiangxi Province, the Ganzhou Basin is a result of Mesozoic extensional tectonic activity and thus shows a “domino-style” stacking pattern. During the annual excursion led by the Shishang Museum of Natural History in 2011, the specimen SMNH-20140105 was discovered in the southwestern part of the Ganzhou Basin by one of the authors (T.-R.Y.) that is filled with dark red purplish sandstone with interbeds of conglomerate (Bureau of Geology and Mineral Resources of Jiangxi Province 1984; He et al. 2017).

**Fig. 4:**
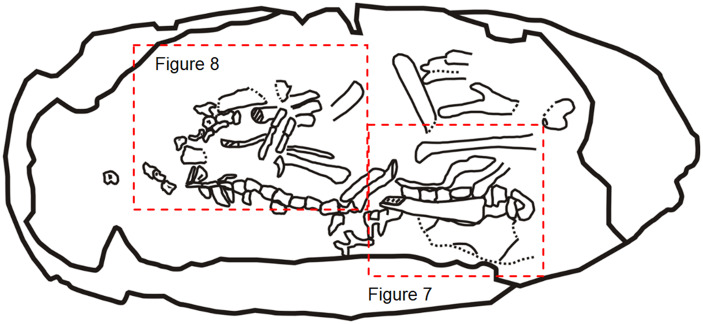
Interpretative drawing of Egg III (SMNH-20140105-III).

After its discovery, the specimen SMNH-20140105 was acquired by the Shishang Museum of Natural History in 2014 for further preparation and studies. All three eggs were part of the same block when collected, as shown in Fig. 2A. Meticulous preparation of each egg was only possible after separating them. The preparators at SMNH and one of the authors (T.-R. Yang) conducted separation and preparation. Each egg was prepared utilizing a micro-sandblaster and air scribes at the SMNH (Fig. 2A). Although the stratigraphic orientation of SMNH-20140105 was not recorded in the field, the specimen contains identifiable geopetal features that indicate the original orientation which is crucial for our further interpretation (see “Results” section).

To access the developmental stage of each embryo-containing egg, we sampled the eggshell of the middle part of each egg for thin sectioning. The eggshell thin sections were examined with a Leica DMLP Polarizing Microscope. Normal and polarized light images were acquired with a Leica DFC420 camera by using the Leica ImageAccess EasyLab 7 software. The calcium of the eggshell is a predominant resource for eggshell formation so that the eggshell is absorbed from the innermost eggshell during embryogenesis, which provides information on the developmental stage of the eggs (Cheng et al. 2008; Weishampel et al. 2008).

For producing photogrammetric 3D models for each embryo-containing egg (Supplementary Figs. 1–3), digital photos (ca. 200 shots per egg) were taken with a SONY DSC-RX100M2 camera and processed using Agisoft PhotoScan Professional version 1.3.1 (www.agisoft.ru/products/photoscan/professional/).

To obtain additional osteological information, Egg III (SMNH-20140105-3; Figs. 2D and 4) was scanned using X-ray computed tomography (CT) at the Institute of Geosciences, University of Bonn (Bonn, Germany) and neutron tomography at the Heinz Maier-Leibnitz Zentrum of the Technische Universität München (TUM) in Garching, München, Germany. The X-ray CT scan was obtained with a Phoenix v|tome|x s (GE Phoenix X-ray; 240 kV) with a voltage of 170 kV and a current of 130 μA. The 800 images were obtained at a voxel size of 0.18528842 mm, each with an exposure time of 400 ms. Three-dimensional reconstructions and measurements were generated using Avizo version 8.1. Neutron radiography and tomographic studies were performed at two imaging beamlines available at the Heinz Maier-Leibnitz Zentrum of the Technische Universität München (TUM) in Garching, München, Germany: the fission and thermal neutron radiography and tomography facility NECTAR and the cold neutron imaging instrument ANTARES. The specification of the NECATR and ANTARES imaging beamlines can be found elsewhere (Bücherl and Söllradl 2015; Schulz and Schillinger 2015; Mühlbauer et al. 2018). The images (radiographs, 2- and 3-D-tomographs, etc.) obtained from probing objects by means of neutron imaging often show complementary or additional information compared to the investigation with X-rays or γ-radiation. In our case, the high penetration power of neutrons allowed us to get insight into the internal structure of the specimens, which was not possible with X-rays. Low energy X-rays could not penetrate such large specimens, while high energy X-rays do not provide sufficient contrast between skeleton and the surrounding material. The measurements at the NECTAR and ANTARES facilities were performed using similar detector setups composed of a scintillator ^6^LiF/ZnS screen, which converts captured neutron radiation into visible light, optical lens and a CCD camera that converts the light into digital images. The size of the scintillator and optics settings available at NECTAR provide a very large field of view (about 30 cm × 30 cm) allowing for scanning a whole specimen at once. The field of view used at ANTARES was slightly smaller (about 20 cm × 20 cm), however cold spectrum (ANTARES) was expected to provide more details of the inner structure of the eggs that are not visible with thermal neutrons (NECTAR). On the contrary, thermal neutrons could better penetrate each egg, which was a challenge with cold neutrons resulting in some artifacts in the reconstructed 3D images of specimens. Therefore, neutron CT was performed at both instruments. Reconstruction of the tomographic scans was performed using commercial Octopus Imaging Software (Version Accessed: 09-Aug-2017).

## Results

### Taxonomic and ootaxonomic inferences

The Nanxiong Group (treated as a formation in some of the references cited here), in which the specimen SMNH-20140105 was discovered, has yielded a great number of oviraptorid dinosaurs, including *H.huangi* (Lü 2002), *Shixinggia oblita* (Lü and Zhang 2005), *Banji long* (Xu and Han 2010), *Ganzhousaurus nankangensis* (Wang et al. 2013), *Nankangia jiangxiensis* (Lü et al. 2013a), *Jiangxisaurus ganzhouensis* (Wei et al. 2013), *Huanansaurus ganzhouensis* (Lü et al. 2015), *Tongtianlong limosus* (Lü et al. 2016), and *Corythoraptor jacobsi* (Lü et al. 2017). Despite numerous contemporaneous oviraptorid dinosaurs unearthed from the same formation, none of them has been associated with an ootaxon except for two oviraptorid embryos of *H.huangi* (NMNS-0015276-F02-embryo-01 of Cheng et al. 2008).

In *Heyuannia*, the femoral shaft is curved anteriorly (Lü 2002); a slight curvature is also observed in the femur of Egg III. The tibia length to femur length ratio is 1.25 in *Heyuannia* (Lü 2002), 1.24 in *Nomingia* (Barsbold et al. 2000), 1.10 in *Wulatelong* (Xu et al. 2013), 1.19 in *Corythoraptor* (Lü et al. 2017), 1.05 in *Nankangia* (Lü et al. 2013b), 1.28 in *Tongtianlong* (Lü et al. 2016), 1.25 in *Anzu* (Lamanna et al. 2014), and 1.18 in *Chirostenotes* (Currie and Russell 1988); in non-oviraptorid theropods, it is 1.23–1.31 in *Compsognathus*, 1.09 in *Deinonychus*, 0.71 in *Ornitholestes*, and 0.99 in *Dilophosaurus* (all measurements from Currie and Russell 1988). In Egg III, the ratio of tibia length to femur length is approximately 1.20.

Proximally, metatarsals II, III, and IV of the Egg III embryo are not co-ossified, as is the case in the *Heyuannia* metatarsals (Lü 2002). The Egg III embryo also shows a long and slender tibia and a curved and robust femur, which can be found in adult oviraptorosaurians (Funston et al. 2018). Since the skull is absent or unexposed in all eggs of the studied clutch SMNH-20140105, these specimens are here considered as Oviraptoridae *incertae sedis*, pending new information.

In comparison with other previously described embryo-containing eggs, the shell of Egg III shows similar ratios between the thickness of the continuous layer (CL) and mammillary layers (MLs) as well as microstructural features to the specimen NMNS-0015276-F02-embryo-01 (Cheng et al. 2008). In addition, the specimen NMNS-0015276-F02-embryo-01 (Cheng et al. 2008) and the three embryos (Wang et al. 2016) were discovered in the same formation, though in adjacent localities (around 30 km away). However, Wang et al. (2016) did not assign the specimens IVPP V20182, IVPP V20183, and IVPP V20184 to any ootaxa. Therefore, based on the egg shape (Tables 1 and 2), histological and surficial features of eggshell (Fig. 2B and D; Table 2), and the geographical affinity, we tentatively assign these eggs to *Macroolithus* sp., which is probably laid by oviraptorids (Cheng et al. 2008; Wang et al. 2016).

**Table 1:** Dimensions of each egg of the specimen SMNH-20140105

Catalog number	Length (mm)	Width (mm)	Factor of compaction (%)
Egg I (SMNH-20140105-1)	184	82	30–32
Egg II (SMNH-20140105-2)	145	75	29–32
Egg III (SMNH-20140105-3)	165	74	30–33

**Table 2:** Parameters of eggshell microstructures in comparison with previously reported specimens

Catalog number	Egg length (mm)	Egg width (mm)	Thickness of CL (mm)	Thickness of ML (mm)	Ratio of CL to ML	Ootaxonomic assignment	Reference(s)
SMNH-20140105-3	165	74	1.25–1.45	0.3–0.4	3.6–4.1	*Macroolithus yaotunensis*	This study
IGM 100/979	180	65					Norell et al. (1995)
IVPP V9608	150	55	0.55	0.2	3.7		Dong and Currie (1996)
IGM 100/971	120	60^a^			3.7	Elongatoolithidae indet. or *Elongatoolithus elongatus*	Norell et al. (2001)
MPC-D100/1017			0.48	0.19–0.33	1.5–2.5	Elongatoolithidae indet.	Weishampel et al. (2008)
NMNS-0015276-F02-embryo-01	175.3	92.1	0.96–1.35	0.24–0.44	3.1–4	*Macroolithus yaotunensis*	Cheng et al. (2008)
MPC-D100/1017			0.48	0.19–0.33	1.5–2.5	Elongatoolithidae indet.	Weishampel et al. (2008)
IVPP V20182	198.3	88.0				Elongatoolithidae indet., but very possibly *Macroolithus yaotunensis*	Wang et al. (2016)
IVPP V20183	179.5	92.1	0.96–1.32	0.24–0.44	3.3–4	Elongatoolithidae indet., but very possibly *Macroolithus yaotunensis*^b^	Wang et al. (2016)
IVPP V20184	163.5	74.8				Elongatoolithidae indet., but very possibly *Macroolithus yaotunensis*^b^	Wang et al. (2016)

a Estimated value.

b Personal communication with the authors of Wang et al. (2016).

### Geopetal features and implications from oviraptorid clutch architecture

Numerous oviraptorid clutches uncovered from China and Mongolia elucidated their unique clutch architecture and revealed geopetal criteria. In comparison with well-preserved clutches (e.g., Yang et al. 2019), specimen SMNH-20140105 represents a partial clutch (Fig. 2A). The paired eggs (Eggs II and III) represent siblings from the same oviposition (Sato et al., 2005). However, the temporal relationship can be only deduced by geopetal features in combination with the developmental stage of each egg, provided that they were not affected by post-mortem manipulation or taphonomic disturbance.

All three eggs were so heavily compacted that the blunt ends are not identifiable simply based on the geometrical shape. However, *Macroolithus* eggs have a blunt end that is covered by lineartuberculate ornamentation, while the acute end lacks ornamentation (Zhao 1975); the attitude of each egg can thus be identified. Post-mortem manipulation or taphonomic disturbance is hence considered unlikely since all blunt ends point to the same direction (Fig. 2A; see the 3D models in Supplementary Figs. 1–3).

The specimen provides three geopetal features that allow for a convincing identification of top and bottom. The first line of evidence is the orientation of the blunt end of an egg. In oviraptorid clutches, all eggs are arranged with their blunt ends pointing upwards and to the center devoid of eggs (Norell et al. 1995, 2018; Sato et al. 2005; Pu et al. 2017; Tanaka et al. 2018; Yang et al. 2019). The second geopetal feature is the stacking pattern of the three eggs. In a well-preserved oviraptorid clutch that consists of several rings of eggs (Norell et al. 1995, 2018; Sato et al. 2005; Pu et al. 2017; Tanaka et al. 2018; Yang et al. 2019), the rings increase in diameter from lowest to highest. Based on the location of their blunt ends, Eggs II and III are closer to the center of the clutch than Egg I and thus are from a lower ring. The third feature is the location of the embryonic remains in each egg, which are expected to gravitationally accumulate at the bottom of the egg after death and disarticulation, coming to rest right atop the eggshell (Sander et al. 2008). The embryonic bones were not well visible in the specimen, though all of them did not preserve eggshell on the top side prior to further preparation and separation into the single eggs (Fig. 2A). The embryonic bones were revealed by removing the sediment from the exposed side in all three eggs, thus indicating that the bones accumulated on the unexposed side and thus must represent the lower side of the eggs (field-bottom). Based on these lines of evidence, we can reconstruct the stacking pattern and be assured that the paired eggs (Egg II and Egg III) were laid prior to the single egg (Egg I).

### Shell histology

Embryo-induced erosion is commonly observed in avian eggs (Orłowski and Hałupka 2015) and was used as an indicator for the developmental stage of the embryo (Cheng et al. 2008; Weishampel et al. 2008; Orłowski and Hałupka 2015; Wang et al. 2016). All radial sections of the eggshells from Egg I, Egg II, and Egg III exhibit a distinct undulating boundary between the CL and ML (Fig. 2E–G; Table 2). The thickness ratio of the CL to the ML ranges from 3.57 to 5.16 (Fig. 2E–G; Table 2). Based on the proportions of the remaining mammillary cone, we estimate that the ratio of CL to ML in Egg III was 0.28–0.33 before resorption of the ML (Table 2). The absence of all mammillary tips in Eggs II and III indicates significant calcium removal from the innermost shell by the developing embryos (Cheng et al. 2008; Weishampel et al. 2008; Orłowski and Hałupka 2015; Wang et al. 2016). Thus, the comparison of mammillary intactness between Egg I and Egg II/III suggests that Egg II/III is more developed than Egg I (Fig. 2E–G).

### Osteological features

**Fig. 5:**
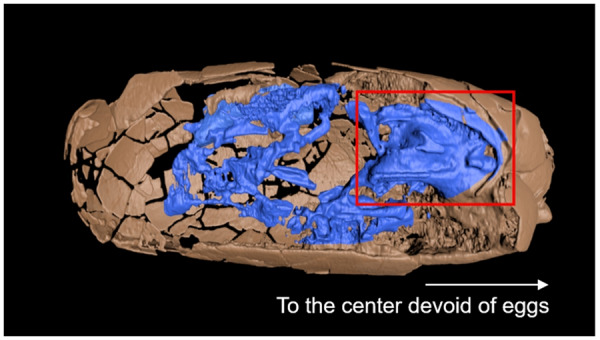
Reconstructed X-ray CT image of Egg III (SMNH-20140105-3). The aggregation in the red box probably represents the cranial parts of the embryo, indicating the pose of the embryo inside the egg. The white arrow points to the center of the original clutch, which is not observable now.

**Fig. 6:**
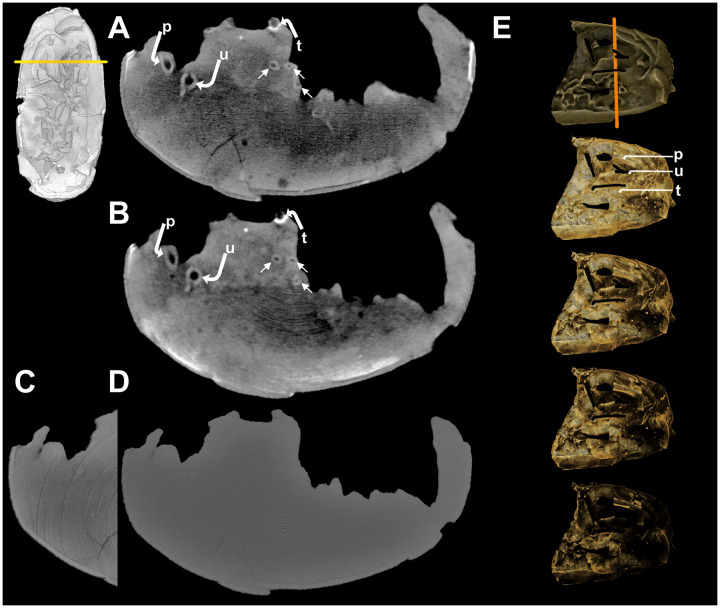
A comparison of the different tomographic imaging techniques used in this study using Egg III (SMNH-20140105-3). A cross section along the orange line of Egg III from (**A**) ANTARES, (**B**) NECTAR, and (**C** and **D**) X-ray CT with different contrasts. The arrows point out the cortical rim of the embryonic long bones in Egg III. (**E**) Volume renderings based on NECTAR with different thresholds for the visibility of different materials and their densities. p, pubis; t, tibia; u, unknown.

While X-ray CT failed to reveal internal skeletal remains of the eggs at sufficient spatial resolution and contrast between bones and sediment (Fig. 5), our study, for the first time, reveals the capability of neutron CT to identify embryonic long bones *in ovo* in fossils (Fig. 6). Tomographic images from both NECTAR and ANTARES show “rims” that represent the cortical part of preserved embryonic remains and are used for bone segmentation (Fig. 6).

#### Egg I (SMNH-20140105-1)

Based on the deformed shape viewed from the polar, which should have been a circle, the factor of compaction is estimated to be 30–35% (Yang et al. 2019). The embryo skeleton is disarticulated. Three metatarsal bones and scattered vertebrae are recognizable but not osteologically assignable. Morphologically, Egg I is 10–20% larger than Eggs I and II (Table 1), suggesting that Egg I and the pair (Egg II/Egg III) were possibly laid by two different females of different body sizes because egg size is constrained by the size of the female pelvis (Shatkovska et al. 2018).

#### Egg II (SMNH-20140105-2)

Eggs II and III are similar in size and factor of compaction. To keep Egg II intact, only a partial mechanical preparation was carried out that only revealed scattered vertebrae. These vertebrae are recognizable as such but ill-defined.

#### Egg III (SMNH-20140105-3)

**Fig. 7:**
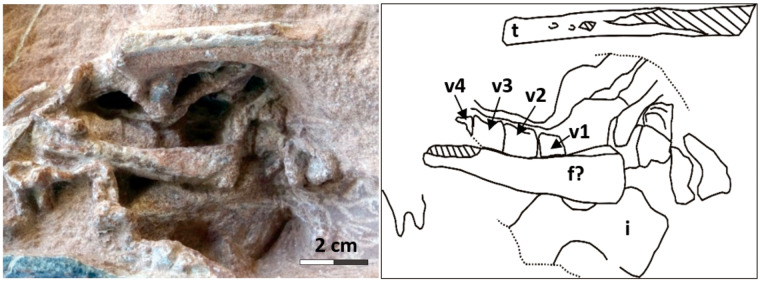
Pelvic region of the oviraptorid embryo in the Egg III. Scale equals 2 cm. f, femur; I, ilium; v, vertebra; t, tibia.

**Fig. 8:**
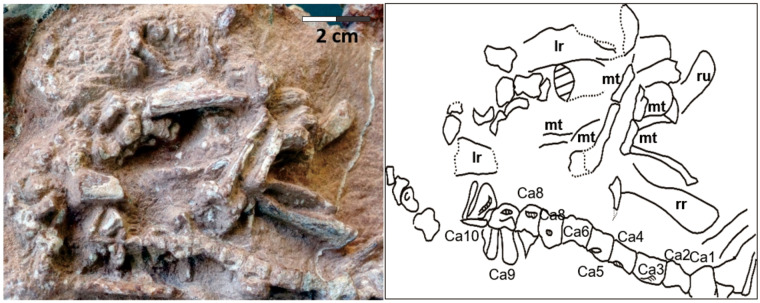
Caudal region of the oviraptorid embryo in the Egg III. Scale equals 2 cm. Ca, caudal centrum; mt, metatarsal; lr, left radius; rr, right radius; ru, right ulna.

Egg III preserves numerous recognizable postcranial remains (Figs. 2D and 4). The neutron CT scan revealed a bone aggregation near the blunt end, possibly representing the cranial part of the embryo (Fig. 5). However, cranial parts were not exposed by preparation. Neutron imaging or synchrotron tomography might be able to resolve the possible preservation of the cranial parts in the matrix. The sacral region is possibly represented by four vertebrae (v1–v4) exposed in the lateral view close to the possible ilium (Fig. 7). However, sacral ribs are not recognizable. A total of 15 caudal vertebrae (Ca1–Ca15) are preserved in lateral view in a continuous series, extending to the acute end of the egg in Egg III (Ca1–Ca15 in Fig. 8). The size of the caudal vertebrae decreases from Ca1 to Ca15, indicating that the Ca15 is the posteriormost of these vertebrae. The neural arch is absent in all caudal vertebrae except for Ca9, possibly due to poor preservation or incomplete ossification. Right next to the series of caudal vertebrae, there is an aggregation of a right ulna, two radii, and scattered metatarsals. The right ulna is identified based on its unmatured distal styloid process and its relative position in relation to the other skeletal remains. Both radii are recognizable since they present distinct styloid processes at their distal ends. The left radius possibly broke during fossilization. The distal part of the right femur is recognizable on the basis of its shape in lateral view; however, the head of the proximal embryonic femur is not well defined. The proximal part of left femur is also visible, an interpretation based on the proximity of the bone to the ilium and more pronounced shape of the head. However, the length of both femora cannot be measured. The distal part of a tibia in posterior view can be identified as well, based on the medial malleolus. Only the proximal halves of the left metatarsals are preserved in Egg III.

While the cranial parts of the embryo in Egg III are not visible, the pose of the embryo inside the egg is indicated by the orientation of the caudal vertebrae (Figs. 4 and 8) which indicates that the cranial parts of the embryo were near the blunt end of the egg, that is, in the direction of the center of the clutch.

## Discussion

### Embryo position *in ovo*

The *Beibeilong* embryo reported by Pu et al. (2017) is a helpful analog to interpret the position of oviraptorid embryos *in ovo*. Oviraptorosaurian embryos exhibit a similar position *in ovo* to modern chickens, with cranial parts toward the blunt end of the egg and caudal end toward the acute end. Although the cranial part of the embryo is not exposed by mechanical preparation, we are able to identify the *in ovo* position of the embryo based on the direction of the caudal vertebral column (Figs. 4 and 8). Furthermore, the neutron CT image of Egg III reveals an aggregate of skeletal remains near the blunt end, possibly representing the cranial part of the embryo (Fig. 5). The probable embryonic skull located near the blunt end indicates that the embryo would have to hatch from the blunt end. This observation concurs with the hypothesis that oviraptor clutches were partially exposed to the air, but with the acute ends buried in substrate (Yang et al. 2019).

### Hatching asynchrony inferred from SMNH-20140105

Hatching asynchrony can be inferred if embryos of different developmental stages are present in a single clutch, provided that (1) the embryos developed continuously and at the same rate, (2) there was no developmental arrest, and (3) there were no heterochronous premature deaths. The developmental arrest is unlikely based on the inference from the extant phylogenetic bracketing of birds and crocodilians (Williamson et al. 2017). The presence of different developmental stages can be determined based on the size of skeletal elements and histological examinations (Weishampel et al. 2008). However, these methods cannot be applied in the present specimens since they do not preserve the same skeletal parts. Instead, our inference of hatching asynchrony is mostly based on the level of articulation.

Norell et al. (1995) suggested that the brooding oviraptor and its egg clutch were suffocated to death in a sandstorm. Frequent flood events inferred from the Late Cretaceous fluvial deposit in Jiangxi, China (Bureau of Geology and Mineral Resources of Jiangxi Province 1984; He et al. 2017) contributed to the death of the studied clutch in a similar way. Therefore, the studied embryos might have perished almost synchronously rather than in heterochronously.

A taphonomic explanation for the observed level of articulation is here considered unlikely since all eggs have similar factors of compaction and were preserved in the same block, which represents the same taphonomic history and deformation. While having experienced the same taphonomic history and deformation, the Egg II is much shorter in length than the Egg III (∼20 mm). In fact, the length of a fossil egg is only shortened in the presence of an angle between the egg long axis and compaction force. Numerous oviraptorid egg clutch showed that the eggs are arranged with an angle to the horizon (Norell et al. 1995, 2018; Dong and Currie 1996; Clark et al. 1999; Grellet-Tinner et al. 2006; Fanti et al. 2012), and such an angle could have reached over 40° (Yang et al. 2019). We surmise that their different lengths might be a result of their different original inclinations.

Kundrát et al. (2007) categorized therizinosauroid embryos into four stages based on the level of ossification and the degree of aggregation of skeletal remains. The first stage consists of poorly articulated skeletal remains that are clustered in an aggregation in one corner of the egg. Kundrát et al. (2007) interpreted the aggregation as a result of the confinement by the yolk. However, the aggregation could also be a result of gravitational accumulation after death and disarticulation, which was not considered by Kundrát et al. (2007).

Whereas an aggregation was not observed in our studied specimen, we observed that all embryonic remains sit near the bottom of the eggs, indicating a gravitational accumulation instead of a confinement by the yolk. Hamburger and Hamilton (1951) subdivided the developmental history of a chicken embryo into 46 stages over 20–21 days. Embryological studies have revealed that the anterior vertebral column develops earlier than the posterior part (Diwan and Dhakad 1995). Somite formation reaches the tip of the pygostyle at Stage 22 (3.5 days). In Egg III, the caudal vertebrae are articulated and well developed, indicating that the embryo was near the hatching stage. Egg II, which should have presented similar disarticulation level and number of bones as Egg III does, only showed a few skeletal remains possibly because of our partial preparation. Egg I only preserves scattered vertebrae that are ill-defined, either because of poor ossification or preservation. The latter possibility is here considered unlikely for reasons given above. Egg III was prepared from both sides, ensuring the best possible exposure of all embryonic remains. The one preserved metatarsal in Egg I (0.9 cm) is slimmer than all of the metatarsal bones in Egg III (1.1 cm), supporting our interpretation that Egg III is more developed than Egg I (Fig. 2). The observation of different developmental stages in the three eggs, which is largely based on a greater degree of embryo-induced erosion in the pair (Eggs II and III) and the slimmer appearance of the embryo metatarsal in Egg I compared to the one in Egg III, demonstrates a temporal gap between the two eggs in the inner (lower) ring and the one in the outer (upper) ring. Our study thus provides supportive evidence to Yang et al.’s (2019) observation that oviraptorid dinosaurs laid the innermost, lower ring of eggs first, covered this ring with substrate, and then laid the second ring, and even third ring, of eggs. However, due to the lack of matching skeletal parts, an estimation of the length of the gap between the two laying events in our specimen (Egg I vs. Eggs II and III) is not possible.

### Hatching asynchrony as a synapomorphy of oviraptorids

Weishampel et al. (2008) described four oviraptorid embryo-containing eggs, possibly from the same clutch but refrained from assigning them to a particular species due to the immaturity of the bones and poor preservation. Based on the assumption that the four embryo-containing eggs were from the same clutch, they hypothesized hatching asynchrony in oviraptorids. The four embryo-containing eggs were discovered in the Nemegt Formation of Bugin-Tsav, Mongolia, which was tentatively dated to a 72.0–70.8 Ma interval (late Campanian; Ogg et al. 2004). Oviraptorids from Mongolia include *Ingenia* sp., *Rinchenia mongoliensis*, *Avimimus nemegtensis*, *Nomingia gobiensis*, and cf. *Elmisaurus* (and possibly other unknown species), all of which have been described from the Nemegt Formation in Bugin-Tsav, Mongolia (reviewed in Funston et al. 2018), indicating a large number of sympatric species. Our specimen, likewise, could potentially pertain to any of a number of species reported from Nanxiong Group (Campanian—Maastrichtian) of Jiangxi and Guangdong Provinces, China. If the hatching asynchrony reported by Weishampel et al. (2008) can be confirmed, hatching synchrony may be a shared character among Oviraptoridae. The latest phylogeny of modern birds on the basis of genomic analyses shows that hatching asynchrony is only observed in Neoaves (Stoleson and Beissinger 2010; Prum et al. 2015). All sauropods are considered to lay a whole clutch *en masse* (Reisz et al. 2012), and the eggs from the same clutch hatched synchronously as in modern reptiles in the absence of incubation behaviors. Among theropods, so far, Troodon eggs have been suggested to hatch synchronously (Varricchio and Jackson 2003). The evidence from our study demonstrates the earliest hatching asynchrony within the dinosaurian clade.

### Thermoregulatory contact incubation and communal nesting behaviors

Previous studies suggested that oviraptorid dinosaurs were paternal-caring, polygamous, communal nesters (Varricchio et al. 2008; Moore and Varricchio 2016). Since oviraptorid dinosaurs were close relatives of modern birds and already showed many of the distinctive avian reproductive features (Varricchio and Jackson 2016), it is very likely that—if contact incubation was present—their embryos only started development after incubation had commenced and that they only started incubation after clutch completion as modern communal nesting birds do (Vehrencamp 1977; Riehl and Jara 2009; Riehl 2010). However, the possible occurrence of thermoregulatory contact incubation in oviraptorid dinosaurs, as has been inferred from adult-associated clutches, is still under debate (Deeming 2002).

The hatching asynchrony in oviraptorids inferred from our study represents a new line of evidence for the interpretation of the adult-associated clutches and has the following implications. First, if oviraptorids did utilize thermoregulatory contact incubation, then oviraptorids were analogous to some modern birds such as barn owls that exhibit extreme hatching asynchrony (Wilson et al. 1986). In combination with the hypothesis that oviraptorids were communal nesters and the male was in charge of incubating the whole clutch (Varricchio et al. 2008; Moore and Varricchio 2016), the male may have started incubation before several females completed the clutch. However, this has not been observed in any modern polygamous bird.

**Fig. 9:**
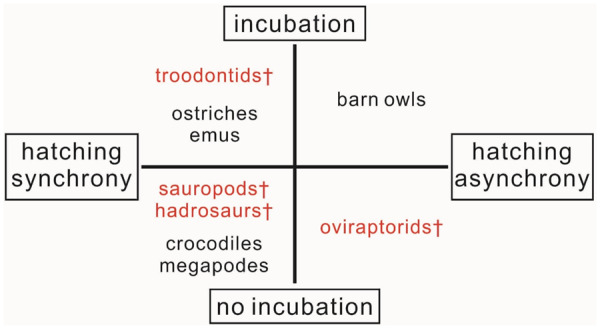
The four possible combinations of incubation mode and hatching mode in extinct clades such as sauropods, hadrosaurs, oviraptorids, and troodontids, all of which are marked in red, and modern crocodiles and birds.

Second, if there was no or ineffective thermoregulatory contact incubation in oviraptorids, the hatching asynchrony may be a result of the time interval between different ovipositions or egg rings because an effective thermoregulatory contact incubation after clutch completion would have synchronized all eggs’ hatching. However, the multiple-ring arrangement in oviraptorid clutches could have evolved because of selective advantages of hatching asynchrony. This peculiar strategy is not observed in any modern animal (Fig. 9), which suggest that the nesting behaviors of oviraptorids are not analogous to those of any modern animals and that caution is therefore warranted when including oviraptorids in analyses of the ancestral state of bird reproduction.

As contact incubation can probably be excluded in oviraptorid dinosaurs (Yang et al. 2019), the second inference is preferred here. Amiot et al. (2006) and Eagle et al. (2015) also partly supported the second inference by suggesting a lower incubation temperature than in modern birds. Although we precluded the first inference, the second inference is still pending validation. Since shell microstructure is a reliable indicator of the developmental stage of the embryo, the level of resorption shown by the shell of the individual eggs of a clutch could potentially test this possibility. More practically, a more complete clutch with embryos would allow to test our hypothesis.

## Conclusions

Oviraptorids are a bizarre group of dinosaurs, whose nesting behavior remains challenging to understand despite many well-preserved specimens. The partial clutch containing embryonic remains reported in this study provides further support for the hypothesis of hatching asynchrony in oviraptorid dinosaurs. Despite the uncertainty about the precise taxonomic assignment within Oviraptoridae, this specimen from southern China demonstrates that the hatching asynchrony of modern birds can be phylogenetically traced far back to oviraptorid dinosaurs and may be a shared derived character of oviraptorid dinosaurs. The pose of the embryo in our study indicates that the embryo hatched from the exposed blunt end where the center of the clutch would have been. This observation concurs with the reconstruction of a partially open oviraptorid clutch based on eggshell porosity analysis and clutch architecture. In combination with their surmised communal nesting behavior, the observed hatching asynchrony unfavored the hypothesis of thermoregulatory incubation behavior in oviraptorids and very possibly represent a result of the time interval between different ovipositions. In conclusion, oviraptorid dinosaurs exhibited peculiar and unique nesting strategies that are not analogous to those of any modern animal. The peculiar oviraptorid reproductive biology renders a simple dichotomy between a “bird model” and a “crocodile model” to infer behaviors of extinct animals problematic.

## Institutional abbreviations

IGM, Mongolian Institute for Geology, Ulaanbaatar, Mongolia; IVPP, Institute of Vertebrate Paleontology and Paleoanthropology, Beijing, China; MPC, Mongolia Paleontological Center, Ulaanbaatar, Mongolia; SMNH, Shishang Museum of Natural History, New Taipei City, Taiwan.

## Supplementary Material

obz030_Supplementary_Data
