# Supplementary material for: Hatching Asynchrony in Oviraptorid Dinosaurs Sheds Light on Their Unique Nesting Biology
Source: Integr Org Biol. 2019 Nov 22;1(1):obz030. doi: 10.1093/iob/obz030 (PMC7671163; doi:10.1093/iob/obz030)
Supplement: Supplementary file 1 [file obz030_supplementary_data.pdf]

Egg II

Egg III
